# Endovascular revascularization of heavily calcified occlusion in superior mesenteric artery using Transcollateral approach

**DOI:** 10.1186/s42155-021-00232-8

**Published:** 2021-06-01

**Authors:** Kazunori Horie, Akiko Tanaka, Norio Tada

**Affiliations:** Department of Cardiovascular Medicine, Sendai Kousei Hospital, 4-15 Hirose-cho, Aoba-ku, Sendai, Miyagi 980-0873 Japan

**Keywords:** Superior mesenteric artery, Computed tomography, Transcollateral approach, Endovascular therapy

## Abstract

**Background:**

Mesenteric ischaemia is often a manifestation of severe vascular disease involving the superior mesenteric artery (SMA). Endovascular revascularization is challenging in a chronic total occlusion (CTO) of SMA.

**Case presentation:**

A-73-year-old male patient was referred to our hospital because of a 2-year history of post prandial abdominal angina. Computed tomography (CT) images revealed a heavily calcified CTO in the ostium of SMA and three-dimensional CT (3D-CT) detected pancreaticoduodenal arcade with filling from the celiac artery. Then, endovascular procedure was attempted; however, angiography did not show the collateral route suitable for transcollateral approach. As demonstrated on the CT, we were successful in passing a guidewire through the SMA-CTO via the celiac trunk transcollateral route. After pull-through of the guidewire, two balloon-expandable stents were deployed in the ostium of SMA. During 3 months after stent implantation, the patient had no further episodes of abdominal angina on dual-anti-platelet therapy.

**Conclusion:**

We demonstrate a case of a heavily calcified SMA occlusion successfully treated with endovascular stenting employing a transcollateral approach, guided by 3D-CT.

**Supplementary Information:**

The online version contains supplementary material available at 10.1186/s42155-021-00232-8.

## Background

Mesenteric ischaemia is often a manifestation of severe vascular disease involving the superior mesenteric artery (SMA) and/or the celiac artery. Endovascular therapy (EVT) of these vessels is now an acceptable treatment to improve clinical symptoms (Chahid et al., 2004; Nyman et al., 1998; Landis et al., 2005; Zettervall et al., 2017). It is challenging to recanalize a chronic total occlusion (CTO) of the SMA via a traditional antegrade approach; several case reports have demonstrated the efficacy of the transcollateral approach (TCA) in SMA-CTO via the celiac trunk and the inferior mesenteric artery (Milner et al., 2004; Robken & Shammas, 2007). Previous reports have described the feasibility of preoperative multidetector computed tomography (CT) in demonstrating important details such as vessel sizes, route and distribution of calcification in EVT for CTO in limb arteries (Della Schiava et al., 2020; Hayakawa et al., 2021). We report a case of a heavily calcified SMA-CTO successfully treated via the celiac artery-transcollateral approach, guided by three-dimensional CT (3D-CT) imaging.

## Case presentation

**Fig. 1:**
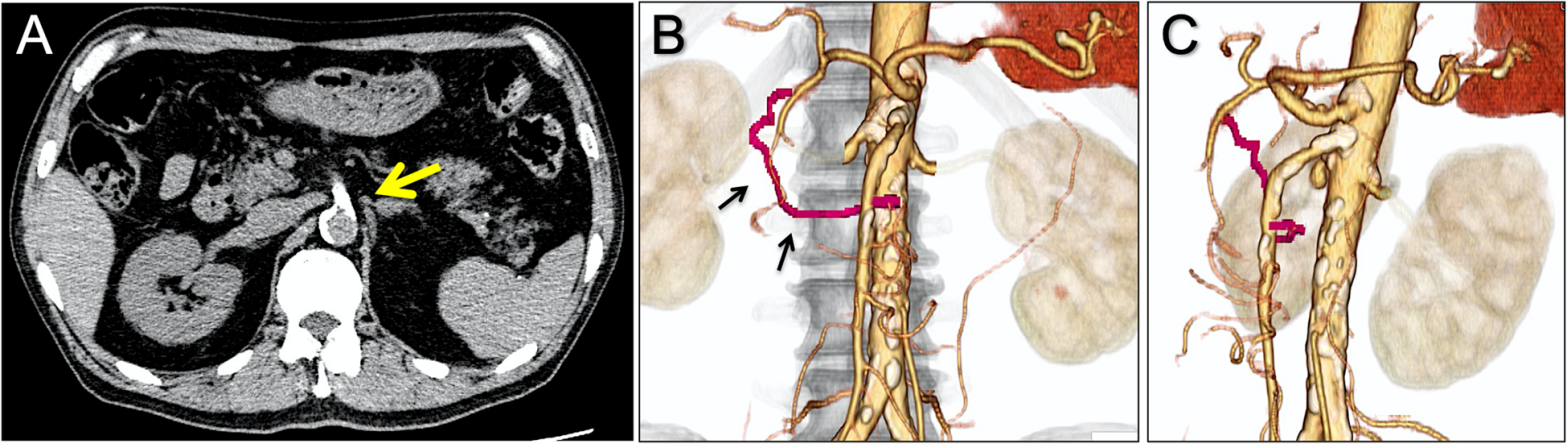
Computed tomography. **A**, Noncontrast computed tomography (CT) revealed a heavily calcified occlusion in the ostium of the superior mesenteric artery (a yellow arrow). **B** and **C**, Contrast-enhanced CT showed a pancreaticoduodenal arcade in the anteroposterior view (black arrows) and in the angulated 65-degree left anterior oblique position

**Fig. 2:**
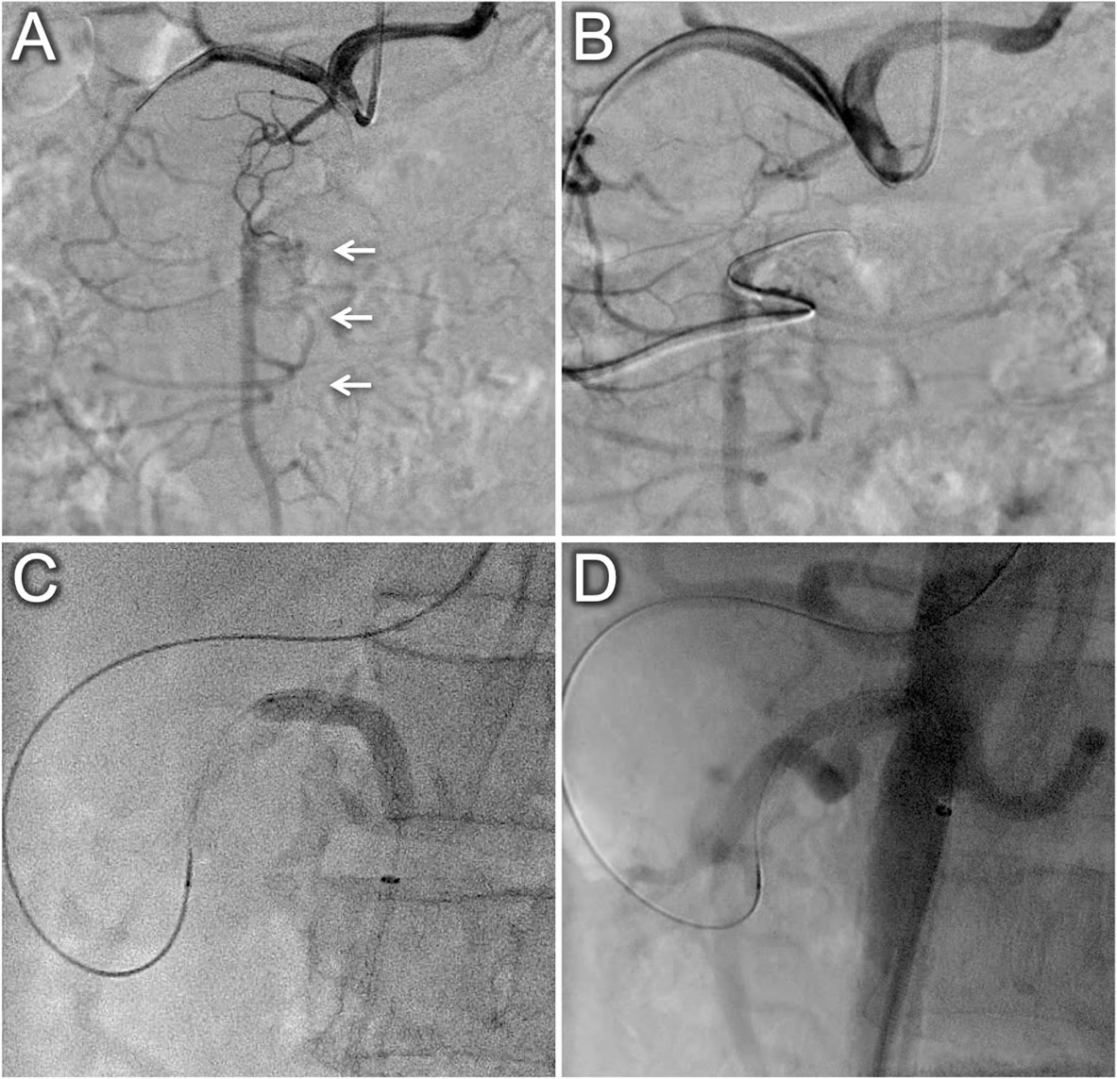
Angiography and endovascular therapy. **A**, Digital subtraction angiography from the celiac artery showed collateral blood flow supplying to the superior mesenteric artery (SMA) (white arrows); however, did not show the pancreaticoduodenal arcade clearly. **B**, The Jupiter SFC guidewire with Corsair microcatheter reached the distal portion of the chronic total occlusion (CTO) in the SMA through the pancreaticoduodenal arcade as shown by computed tomography imaging. **C**, A 6.0 mm balloon was dilatated in the CTO. **D**, Angiography showed antegrade blood flow in the SMA after two balloon expandable stents


A-73-year-old male patient with the history of hypertension, diabetes and lower extremity artery disease was referred to our hospital because of a 2-year history of post prandial abdominal angina. Upper and lower gastrointestinal endoscopies showed no abnormal findings. CT images revealed a heavily calcified CTO at the ostium of SMA (Fig. 1A and axial imaging in Supplementary Movie 1) and 3D-CT demonstrated a patent pancreaticoduodenal arcade with filling of the SMA from the celiac artery (Fig. 1B and C). Angiography of the celiac artery in the anteroposterior view revealed collateral blood flow to the SMA; however, the pancreaticoduodenal arcade was not visualized clearly (Fig. 2A and Supplementary Movie 2). According to the collateral route shown by the 3D-CT, we attempted TCA and retrograde wire crossing of the SMA-CTO. A 6.0-Fr Brite Tip Judkins Right4 guiding catheter (Cordis, Miami, FL, US) via the left radial artery was engaged in the ostium of the celiac artery. We advanced a 150 cm Corsair microcatheter (Asahi Intecc, Aichi, Japan) with a Hi-Torque Command 0.014 guide wire (Abbott Medical, Santa Clara, California, US) into the gastroduodenal artery. A Jupiter SFC guidewire (Boston Scientific, MA, US) was advanced into the superior pancreaticoduodenal artery and to the distal portion of the SMA-CTO. The CTO which was subsequently crossed with a Vassallo 14 guidewire (Cordis) (Fig. 2B). A 6.0-Fr long sheath was inserted into the right common femoral artery and a 12.0–20.0 mm En-Snare (Merit Medical, Tokyo, Japan) was used to capture the Vassallo 14 guidewire, which was withdrawn through the right femoral sheath. Eagle Eye intravascular ultrasound (IVUS; Philips Volcano, Rancho Cordova, CA, US) confirmed the intraplaque wire crossing (Supplementary Movie 3). After dilatation with a 6.0 mm Shiden HP balloon (Kaneka Medix, Osaka, Japan) at 20 atm (Fig. 2C), two 6.0 mm × 18 mm Express Vascular SD stents (Boston Scientific) were implanted in the SMA. IVUS revealed that the stents were well expanded (Supplementary Movie 4), and angiography showed antegrade blood flow in the SMA (Fig. 2D). The patient had no major post-operative complications and was discharged from the hospital. During 3 months after the EVT, the patient had no further episodes of abdominal angina on dual-anti-platelet therapy.

**Additional file 3: Supplementary Movie 3.** Intravascular ultrasound imaging of the superficial mesenteric artery before balloon dilatation.


**Additional file 4: Supplementary Movie 4.** Post-procedural imaging of Intravascular ultrasound in the superficial mesenteric artery.

## Discussion

In patients with mesenteric ischaemia due to chronic SMA occlusion, 2-year primary and secondary patency rates of 76% and 90% respectively, were reported after stent implantation (Zettervall et al., 2017). Despite the slightly suboptimal primary patency rates after stent implantation, EVT for chronic mesenteric ischaemia is associated with lower in-patient mortality and complications compared to open surgery (Zettervall et al., 2017; Oderich et al., 2018). Therefore, EVT for the CTO of SMA and/or celiac arteries is now considered an acceptable and less invasive alternative to surgical revascularization. However, an antegrade approach to treating a SMA-CTO can be difficult as angiography may not identify the origin of the SMA clearly and it may be challenging to catheterise the ostium using guide catheters.

Typically, collaterals to the SMA are frequently seen from the celiac and/or inferior mesenteric artery. Although several reports demonstrated the efficacy of TCA in treating SMA occlusion (Milner et al., 2004; Robken & Shammas, 2007), this is usually performed in well-developed collateral channels. Some patients with SMA occlusions have tiny collaterals between celiac and mesenteric arteries, and angiography may not demonstrate visible targets for treatment via TCA. Wire perforation and arterial dissection in the collateral arteries for SMA can be a fatal complication because of intraabdominal hemorrhage and intestinal necrosis; therefore, reliable routes of TCA should be identified before the EVT. Our case had a heavily calcified CTO and pull-through of a guidewire was necessary to deliver a high-pressure balloon and deploy balloon expandable stents. Although TCA was essential to achieve the revascularization, there are few reports demonstrating the efficacy of CT imaging to evaluate tiny collateral routes in patients with SMA-CTO. In our case, 3D-CT imaging was useful in identifying a viable collateral supply to the SMA via the superior pancreaticoduodenal artery, allowing us to successfully perform our procedure via a TCA.

## Conclusions

We demonstrated a case of heavily calcified CTO in the SMA, in which 3D-CT allowed to visualize the reliable collateral channel from the celiac artery. TCA was safely performed in the guidance of CT imaging and CTO could be treated with stent implantation.

## Supplementary Information

**Additional file 1: Supplementary Movie 1.** Axial imaging of the superficial mesenteric artery assessed by noncontrast computed tomography.

**Additional file 2: Supplementary Movie 2.** Angiography of the celiac artery in the front view.

## Data Availability

The datasets used and/or analyzed during the current study are available from the corresponding author on reasonable request.

## Abbreviations

SMA: Superior mesenteric artery
EVT: Endovascular therapy
CTO: Chronic total occlusion
TCA: Transcollateral approach
CT: Computed tomography
3D-CT: Three-dimensional computed tomography
IVUS: intravascular ultrasound

## References

[CR1] Chahid T, Alfidja AT, Biard M, Ravel A, Garcier JM, Boyer L (2004). Endovascular treatment of chronic mesenteric ischemia: results in 14 patients. Cardiovasc Intervent Radiol.

[CR2] Della Schiava N, Naudin I, Bordet M, et al. (2020) Analysis of preoperative CT scan can help predict technical failure of endovascular treatment of TASC C-D Aortoiliac chronic Total occlusions. Ann Vasc Surg. S0890-5096(20)30789-5 10.1016/j.avsg.2020.08.108 32890648

[CR3] Hayakawa N, Kodera S, Ohki N, Sakkya S, Kanda J (2021). Efficacy of three-dimensional road mapping by fusion of computed tomography angiography and fluoroscopy in endovascular treatment of aorto-iliac chronic total occlusion. Heart Vessel.

[CR4] Landis MS, Rajan DK, Simons ME, Hayeems EB, Kachura JR, Sniderman KW (2005). Percutaneous management of chronic mesenteric ischemia: outcomes after intervention. J Vasc Interv Radiol.

[CR5] Milner R, Woo EY, Carpenter JP (2004). Superior mesenteric artery angioplasty and stenting via a retrograde approach in a patient with bowel ischemia--a case report. Vasc Endovasc Surg.

[CR6] Nyman U, Ivancev K, Lindh M, Uher P (1998). Endovascular treatment of chronic mesenteric ischemia: report of five cases. Cardiovasc Intervent Radiol.

[CR7] Oderich GS, Macedo R, Stone DH, Woo EY, Panneton JM, Resch T, Dias NV, Sonesson B, Schermerhorn ML, Lee JT, Kalra M, DeMartino R, Sandri GA, Ramos Tenorio EJ, Low Frequency Vascular Disease Research Consortium Investigators (2018). Low frequency vascular disease research consortium investigators. Multicenter study of retrograde open mesenteric artery stenting through laparotomy for treatment of acute and chronic mesenteric ischemia. J Vasc Surg.

[CR8] Robken J, Shammas NW (2007). Treatment of a totally occluded superior mesenteric artery facilitated by retrograde crossing via collaterals from the celiac artery. J Endovasc Ther.

[CR9] Zettervall SL, Lo RC, Soden PA, Deery SE, Ultee KH, Pinto DS, Wyers MC, Schermerhorn ML (2017). Trends in treatment and mortality for mesenteric ischemia in the United States from 2000 to 2012. Ann Vasc Surg.

